# Formation of Single‐Holed Cobalt/N‐Doped Carbon Hollow Particles with Enhanced Electrocatalytic Activity toward Oxygen Reduction Reaction in Alkaline Media

**DOI:** 10.1002/advs.201700247

**Published:** 2017-07-06

**Authors:** Bu Yuan Guan, Le Yu, Xiong Wen (David) Lou

**Affiliations:** ^1^ School of Chemical and Biomedical Engineering Nanyang Technological University 62 Nanyang Drive Singapore 637459 Singapore

**Keywords:** hollow structures, MOF composites, oxygen reduction reaction, single‐holed particles, ZIF‐67

## Abstract

Design and construction of metal‐organic framework (MOF) composite precursors have recently been considered as a promising strategy for the preparation of different structured metal/carbon‐based functional materials. Here, an MOF composite‐assisted strategy to synthesize single‐holed cobalt/N‐doped carbon hollow particles is reported. The yolk–shell polystyrene@zeolitic imidazolate framework‐67 (PS@ZIF‐67) composite precursors are first synthesized, followed by a controlled pyrolysis to obtain cobalt/N‐doped carbon hollow particles with a large single hole on each shell. Moreover, the MOF‐coating approach reported in this work can be extended to prepare various core‐shell ZIF‐67 composites with different structures and compositions. Benefiting from the structural and compositional advantages, the as‐derived single‐holed cobalt/N‐doped carbon hollow particles manifest superior electrocatalytic oxygen reduction performance with high activity and excellent durability.

Sustainable electrochemical energy storage and conversion systems have been considered as promising technologies in dealing with the emerging challenges caused by global energy and environmental concerns.^1^, ^2^, ^3^, ^4^, ^5^, ^6^ The oxygen reduction reaction (ORR) is a very important electrochemical reaction for a variety of renewable energy storage and conversion devices, such as fuel cells and metal–air batteries.^7^, ^8^, ^9^ However, the efficiency of these devices has been severely constrained by the sluggish kinetics of the electrocatalytic reduction of oxygen. Currently, platinum (Pt) and its alloys have been regarded as the most active catalysts toward ORR.^10^ Nevertheless, the supply of noble metal like Pt is not sustainable, which makes it unsuitable for large‐scale applications. As a result, continuous efforts have been dedicated to developing efficient and low‐cost non‐platinum catalysts toward ORR. Recently, great advances have been made in enhancing the activity of non‐platinum catalysts to the level comparable to Pt‐based materials.^11^, ^12^, ^13^, ^14^ The key structural feature of this specific family of catalysts is the presence of nitrogen‐coordinated sites with a transition metal, such as Co, Fe, Mn, and Ni, embedded in carbon matrix. These catalysts are usually prepared by pyrolysis of different metal‐organic mixtures consisting of desirable transition metal ions and organic molecules with high‐nitrogen content.^15^ Despite the progress that has been made, it still remains an important research area for design and preparation of highly active catalysts that can reach and even exceed the performance of Pt‐based materials.

In addition to controlling the chemical compositions of the catalysts, their electrocatalytic performance could be further improved by constructing proper structures. Hollow particles with enhanced surface area, shortened distance for mass/charge transfer and reduced aggregation of nanosized subunits, have been widely used as micro/nanoreactors in organocatalysis,^16^, ^17^, ^18^, ^19^ biocatalysis,^20^, ^21^ and electrocatalysis.^22^, ^23^, ^24^, ^25^ In particular, hollow particles with large through‐holes (>50 nm) in the shells have been demonstrated to show great potential in catalytic applications owing to fast mass transport and full utilization of interior catalytic sites.^26^, ^27^ Moreover, controlled formation of sufficiently separated nanosized active sites in the shells of hollow particles with large through‐holes may not only prevent site overlap or catalytic deactivation but also promote the formation and release of formed catalytic products. However, controllable synthesis of hollow particles with sufficiently separated small active sites and large through‐holes in the shells still remains as a great challenge mainly because of the limited strategies for construction of hollow particles with large through‐holes on the surfaces and the severe aggregation of nanoparticles at high reaction temperature.^28^, ^29^

In recent years, synthesis of metal/carbon‐based functional materials derived from metal‐organic framework (MOF) precursors has drawn considerable attention worldwide.^30^, ^31^, ^32^, ^33^, ^34^ Many MOFs constructed from a wide range of transition metal ions and nitrogen‐rich organic ligands are ideal precursors for the synthesis of various electrocatalysts with nitrogen‐coordinated transition metal active sites. However, conventional MOFs‐derived syntheses are mainly based on simple MOF particles, and their derived metal/carbon‐based composite materials often possess solid structures after pyrolysis under inert atmosphere.^35^, ^36^, ^37^, ^38^ Therefore, design and synthesis of MOFs composite precursors by introducing other co‐templates/functional materials into uniform MOF shells may be a simple and versatile way for construction of metal/carbon‐based functional materials with hollow structure and tailored composition.

In this work, we report an MOF composite assisted synthesis of single‐holed cobalt/N‐doped carbon (Co/NC) hollow particles as an efficient electrocatalyst toward ORR. Different from previous works, a uniform layer of ZIF‐67 particles is first grown on polystyrene (PS) spheres to form PS@ZIF‐67 composite particles as the precursor, which is then transformed into hollow particles with a large through‐hole on the surface by a carbonization process (**Figure** **1**). The introduction of the PS sphere into the MOF composite precursor provides a thermally degradable template. The formation of the hole on the surface of Co/NC hollow particle is due to the strong gas outflux of hydrocarbon generated from the decomposition of PS sphere. Furthermore, this strategy can be further applied to grow ZIF‐67 shells on many other functional particles, such as MnO*_x_* nanowires and graphene oxide (GO) nanosheets. Benefiting from the unique shell architecture and robust matrix, the as‐prepared single‐holed Co/NC hollow particles exhibit superior electrocatalytic performance toward ORR.

**Figure 1:**
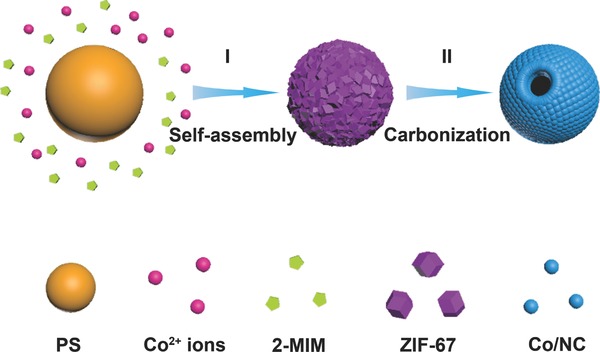
Schematic illustration of the controlled formation of I) PS@ZIF‐67 composite particle(I) and its conversion to single‐holed Co/NC hollow particle by a subsequent thermal treatment (II).

Uniform PS spheres are prepared through a surfactant‐free emulsion polymerization^39^ and their surfaces are further functionalized with polyvinylpyrrolidone (Figure S1, Supporting Information). The growth of MOF shells is carried out by addition of metal source Co(NO_3_)_2_ and organic ligand 2‐methylimidazole (2‐MIM) in the reaction system. In a typical synthesis, PS spheres are mixed with Co(NO_3_)_2_ and 2‐MIM in methanol and then kept at room temperature for 2 h without stirring. The purple product of PS@ZIF‐67 particles is then obtained. Field‐emission scanning electron microscopy (FESEM) images show the morphology of the resultant PS@ZIF‐67 particles (**Figure** **2**a,b). In contrast to large ZIF‐67 single crystals prepared without addition of PS templates (Figure S2, Supporting Information), uniform MOF shells composed of small ZIF‐67 nanocrystals are formed on PS spheres. Transmission electron microscopy (TEM) images reveal that each PS@ZIF‐67 composite particle possesses a yolk–shell structure with the core diameter of about 1.3 µm and the shell thickness of about 210 nm (Figure 2c,d). As revealed by powder X‐ray diffraction (XRD) analysis (Figure S3, Supporting Information), the PS@ZIF‐67 particles show a similar XRD pattern as the large ZIF‐67 crystals. The overall low intensity and broadening of the peaks clearly indicate the nature of the small ZIF‐67 crystals in the MOF shells. The successful growth of ZIF‐67 shells on PS templates can be further confirmed by energy dispersive X‐ray spectroscopy (EDX) measurement (Figure S4, Supporting Information). The distribution of ZIF‐67 particles formed on the surface of PS templates can be tuned by decreasing the concentration of organic ligand (Figure S5, Supporting Information). Despite the capability for the formation of robust and continuous MOF shells on PS templates, segregated ZIF‐67 crystals can be formed on the PS spheres via an island nucleation and anisotropic growth mode by using relatively low concentration of 2‐MIM. More importantly, we further show that many other functional particles with different morphologies, including MnO*_x_* nanowires and GO nanosheets (Figure S6, Supporting Information), can also be encapsulated inside the ZIF‐67 shells to form multifunctional composite materials (Figure S7, Supporting Information).

**Figure 2:**
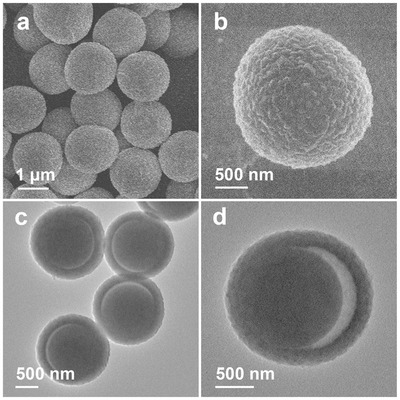
a,b) FESEM and c,d) TEM images of PS@ZIF‐67 particles.

The as‐prepared PS@ZIF‐67 composite particles can withstand the carbonization process under N_2_ atmosphere. The EDX analysis of the pyrolysis product shows the existence of Co, C, and N elements in the sample (Figure S8, Supporting Information). The XRD pattern (Figure S9, Supporting Information) of the product can be indexed to the α‐cobalt phase (JCPDS card No. 01‐1255). No residues or impurity phases are detected, indicating that the PS@ZIF‐67 composite precursor is completely converted to Co/NC composite after the thermal treatment. **Figure** **3**a,b show the FESEM and TEM images of the as‐derived Co/NC hollow particles at low magnification. The sample retains its spherical morphology after carbonization while a unique large hole can be observed in the shell of each particle. Observed from the surface of a Co/NC particle with a large hole (Figure 3c), the crumpled shell and hollow interior of the Co/NC particle can be clearly discerned. Besides the unique large through‐hole, highly porous texture can be clearly observed throughout the whole Co/NC composite shell (Figure 3d). Inspected from the side of the Co/NC particles (Figure 3e,f), the single‐holed hollow structure can be further revealed. A closer examination on the shell of a Co/NC hollow particle reveals that the layer is composed of very small nanoparticles (Figure 3g). High‐magnification TEM observation on the edge of the shell gives more details of the nanostructure. Figure 3h clearly shows that numerous Co nanocrystallites are embedded in carbon matrix. The presence of N‐doped carbon matrix formed from the carbonization of 2‐MIM might play important roles in prohibiting the growth of Co nanocrystallites and stabilizing the hollow particles. A high‐resolution (HR)TEM image (Figure 3i) clearly shows lattice fringes with an interplanar distance of 0.204 nm, corresponding to the (111) planes of α‐cobalt. Judging from their crystal lattices, the size of Co nanocrystallites is typically less than 5 nm. The uniform distribution of Co, C, and N elements is illustrated by elemental mappings shown in **Figure** **4**.

**Figure 3:**
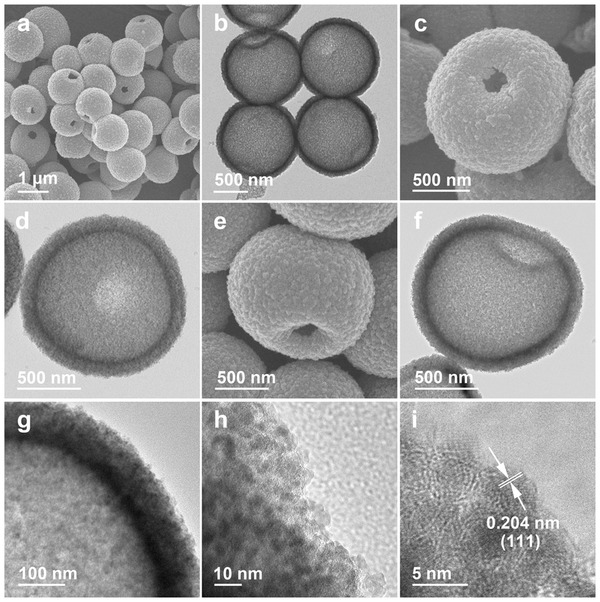
a,c,e) FESEM, b,d,f) TEM, g,h) magnified TEM, i) HRTEM images of single‐holed Co/NC hollow particles.

**Figure 4:**
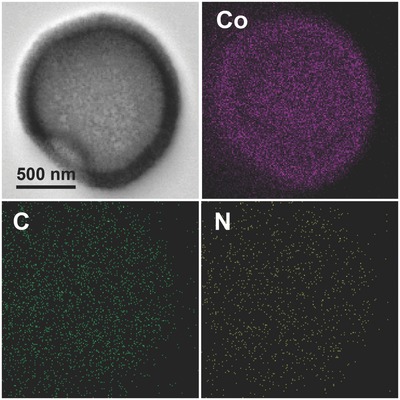
Elemental mappings of a single‐holed Co/NC hollow particle.

It is believed that the strong hydrocarbon gas outflux generated from the thermal decomposition of the PS template leads to the formation of the large through‐hole on the surface of the Co/NC hollow particles. The structural evolution of the Co/NC particles prepared with different heating rates is schematically illustrated in Figure S10a in the Supporting Information. To understand the formation and evolution process, FESEM is used to characterize the products prepared by various pyrolysis processes. By simply increasing the heating rate from 1 to 5 °C min^−1^ and then to 10 °C min^−1^, the structure of the obtained Co/NC particles is continuously varied from hollow particles with small holes, to hollow particles with larger holes, and finally to cracked bowl‐shaped shells (Figure S10b–d, Supporting Information). The opening size of the hole has a correlation with the heating rate in the pyrolysis process. The PS cores in the sample pyrolyzed with higher heating rate are supposed to decompose into hydrocarbon gas more rapidly. As a result, the strong gas outflux generated with the heating rate of 10 °C min^−1^ causes complete rupture of the hollow spheres.

The electrocatalytic activity of the single‐holed Co/NC hollow particles toward ORR is first investigated by cyclic voltammetry (CV) measurements in 0.1 m KOH solution at room temperature. As shown in **Figure** **5**a, no obvious redox peak is observed for single‐holed Co/NC hollow particles in N_2_‐saturated 0.1 m KOH solution. In contrast, when the solution is saturated with O_2_, a pronounced cathodic peak is observed at 0.83 V (vs reversible hydrogen electrode (RHE)). Rotating disk electrode measurements are further used to examine the electrocatalytic activity and kinetics of the single‐holed Co/NC hollow particles in O_2_‐saturated 0.1 m KOH solution (Figure S11a, Supporting Information). The corresponding Koutecky–Levich (K–L) plots (Figure S11b, Supporting Information) reveal the inverse of current density (*j* ^−1^) as a function of the inverse of square root of rotating speed (ω^−1/2^) at different potentials from 0.3 to 0.6 V. The number of electrons involved per O_2_ (*n*) in the ORR for the single‐holed Co/NC hollow particles is determined by the K–L equation. The *n* value is calculated to be about 3.99, suggesting that the ORR is dominated by the four‐electron (4*e*) process and O_2_ is directly reduced to OH^−^. The single‐holed Co/NC hollow particles are benchmarked with a commercial Pt/C electrocatalyst (20 wt%; Johnson Matthey) toward the ORR. Remarkably, the single‐holed Co/NC sample shows higher ORR activity than the commercial Pt/C electrocatalyst in terms of half‐wave potential (0.87 V vs 0.83 V; Figure 5b). Moreover, the electrochemical double‐layer capacitance (*C*_dl_), which is often used to estimate the electrochemical active surface area (ECSA), is determined by measuring the CV curves in the potential range of 1.1–1.3 V without redox processes.^40^ The ECSA is proportional to the *C*_dl_ of a catalyst. Compared with simple Co/NC particles derived from ZIF‐67 particles (Figure S12, Supporting Information), the single‐holed Co/NC hollow particles exhibit much higher *C*_dl_ (Figure S13, Supporting Information). The enhanced ECSA might come from extra active reaction sites on internal surface of single‐holed Co/NC hollow particles. Furthermore, the much smaller Tafel slope of single‐holed Co/NC hollow particles confirms the superior electrochemical kinetics compared to simple Co/NC particles (Figure S14, Supporting Information). Therefore, the excellent electrocatalytic activity may have close relation with the unique structure of single‐holed hollow particles and sufficiently separated small Co nanocrystallites in porous carbon shells. The stability of the single‐holed Co/NC hollow particles and commercial Pt/C catalyst is examined via chronoamperometric responses at 0.5 V in O_2_‐saturated 0.1 m KOH solution with the rotation speed of 1600 rpm (Figure 5c). After the stability test for 24 h, ≈92.9% of the original current density is retained for the single‐holed Co/NC electrode, whereas the Pt/C catalyst electrode shows much higher current loss of 36.5% after only 16 h. The single‐holed Co/NC hollow particles and commercial Pt/C catalyst are further compared by measuring the methanol crossover via chronoamperometric responses at the potential of 0.5 V in O_2_‐saturated 0.1 m KOH electrolyte at the rotation speed of 1600 rpm (Figure 5d). When methanol is added into the electrolyte, there is an instant drop in the current of the commercial Pt/C catalyst electrode. On the other hand, the current of the single‐holed Co/NC electrode remains almost unchanged, which indicates the excellent tolerance for methanol. These results suggest that the single‐holed Co/NC hollow particles show excellent electrocatalytic performance, which is superior to those of many previously reported MOF‐derived catalysts (Table S1, Supporting Information).

**Figure 5:**
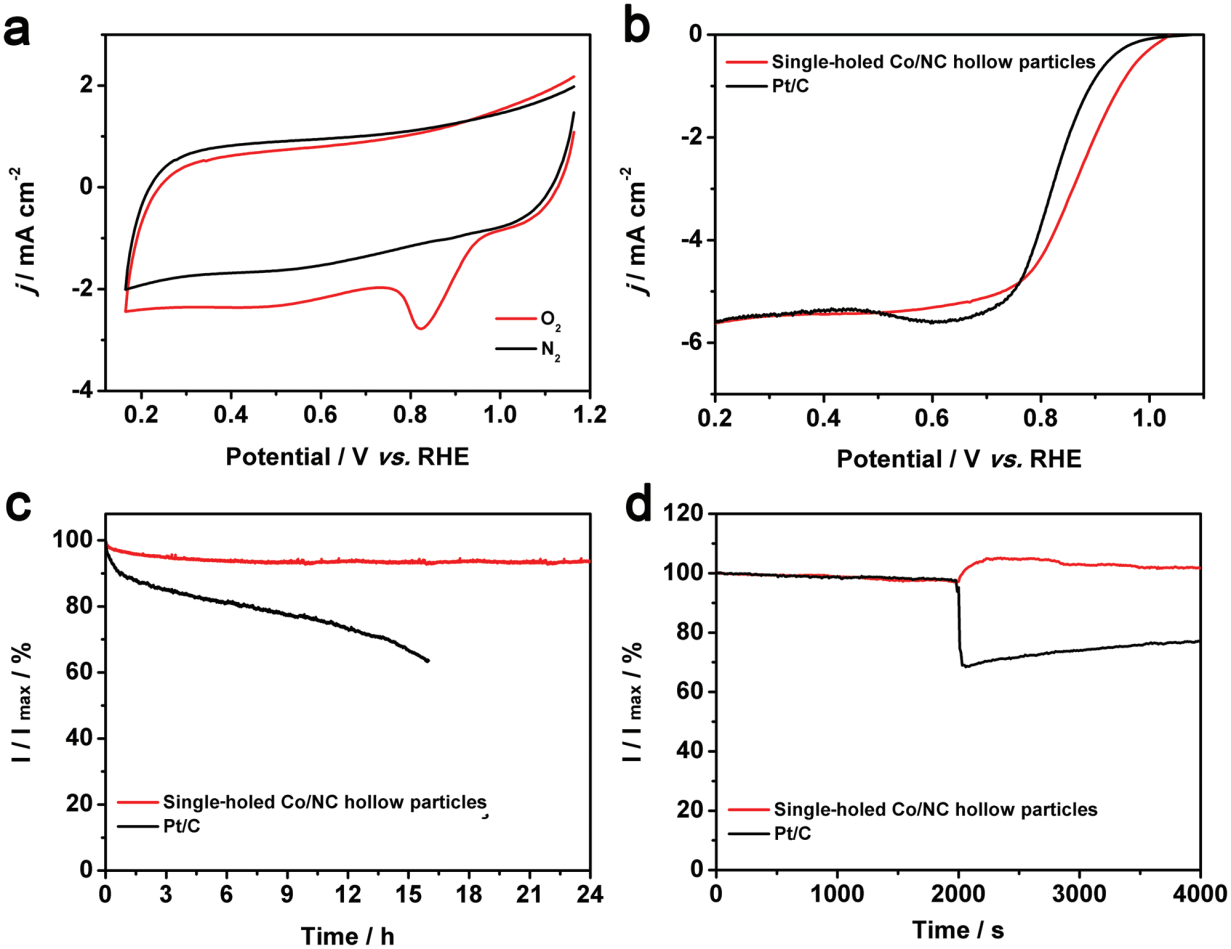
Electrochemical characterizations of single‐holed Co/NC hollow particles as an electrocatalyst for ORR. a) CV curves in N_2_‐saturated and O_2_‐saturated 0.1 m KOH solution with a sweep rate of 20 mV s^−1^. b) Linear sweep voltammetry (LSV) curves of single‐holed Co/NC hollow particles and a commercial Pt/C catalyst in O_2_‐saturated 0.1 m KOH solution with a sweep rate of 10 mV s^−1^ at the rotating speed of 1600 rpm. c) Durability test in O_2_‐saturated 0.1 m KOH at 0.5 V. d) Chronoamperometric responses obtained at 0.5 V in O_2_‐saturated 0.1 m KOH solution (150 mL) with addition of 10 mL of methanol at the time of 2000 s.

In summary, we have prepared unique cobalt/N‐doped carbon nanocomposite hollow particles with a single through‐hole in the shell. The synthesis is realized using polystyrene@zeolitic imidazolate framework‐67 yolk–shell particles as the precursor. During thermal treatment, the PS core in these yolk–shell particles is thermally decomposed to generate gas outflux for the effective formation of large through‐holes on the shells. At the same time, the zeolitic imidazolate framework‐67 shells are easily converted into cobalt/N‐doped carbon microshells composed of separated Co nanocrystallites inside porous carbon matrix. The as‐prepared single‐holed cobalt/N‐doped carbon hollow particles exhibit remarkable electrocatalytic performance toward the oxygen reduction reaction. The superior electrocatalytic properties might be attributed to the unique structure of the obtained single‐holed hollow particles and sufficiently separated electrocatalytic active sites in the microshells. This strategy can be easily applied to prepare other rationally designed metal‐organic frameworks‐derived functional materials for different energy‐related applications.

## Conflict of Interest

The authors declare no conflict of interest.

## Supporting information

Supplementary
