# Supplementary material for: Formation of Single‐Holed Cobalt/N‐Doped Carbon Hollow Particles with Enhanced Electrocatalytic Activity toward Oxygen Reduction Reaction in Alkaline Media
Source: Adv Sci (Weinh). 2017 Jul 6;4(10):1700247. doi: 10.1002/advs.201700247 (PMC5644236; doi:10.1002/advs.201700247)
Supplement: Supplementary file 1 — Supplementary [file ADVS-4-na-s001.pdf]

Copyright WILEY-VCH Verlag GmbH & Co. KGaA, 69469 Weinheim, Germany, 2017.

Supporting Information

for *Adv. Sci.,* DOI: 10.1002/advs.201700247

Formation of Single-Holed Cobalt/N-Doped Carbon Hollow Particles with Enhanced Electrocatalytic Activity toward Oxygen Reduction Reaction in Alkaline Media

*Bu Yuan Guan, Le Yu, and Xiong Wen (David) Lou**

Supporting Information

Formation of Single-Holed Cobalt/N-Doped Carbon Hollow Particles with Enhanced Electrocatalytic Activity toward Oxygen Reduction Reaction in Alkaline Media

*Bu Yuan Guan, Le Yu, and Xiong Wen (David) Lou**

[*] Dr. B. Y. Guan, Dr. L. Yu, Prof. X. W. Lou

School of Chemical and Biomedical Engineering, Nanyang Technological University, 62 Nanyang Drive, Singapore, 637459, (Singapore)

Email: xwlou@ntu.edu.sg; Webpage: http://www.ntu.edu.sg/home/xwlou/

Experimental details

Synthesis of PS particles. The polystyrene (PS) spheres are synthesized according to Ottewill's method (*Colloid Polym. Sci*. **1974**, *252*, 464). In a typical synthesis, 4 ml of styrene (99%, Sigma-Aldrich) is dispersed in 33.5 ml of water, followed by the addition of 2.5 ml of 18 mg ml^{-1} aqueous solution of potassium persulfate ($K_2S_2O_8$, 97%, Alfa Aesar). The mixture is allowed to react under stirring at 70 ^{o}C for 24 h. The as-prepared PS seeds are collected and further dispersed in the mixture of 4 ml of styrene and 33.5 ml of water. The PS spheres with the particle size of about 1.3 μm are produced by repeating the synthetic steps described above again. Then the obtained PS spheres are collected by centrifugation (5000 rpm) and dispersed in 10 ml of ethanol solution of polyvinylpyrrolidone (PVP; 0.5 g, M_w = 40000, Sigma-Aldrich), and the mixture is further stirred at room temperature for 12 h. The PVP-functionalized PS spheres are collected by

centrifugation (7000 rpm), washed by ethanol several times, and dispersed in 30 ml of methanol for further use.

Synthesis of PS@ZIF-67 particles. To synthesize PS@ZIF-67 particles, 0.3 ml of PS spheres suspension and 3 ml of 20 mM $Co(NO_3)_2·6H_2O$ (98%, Sigma-Aldrich) solution are mixed, followed by quick addition of 5 ml of 160 mM 2-methylimidazole (2-MIM, 95%, Sigma-Aldrich) solution. The mixture is then allowed to react at room temperature for 2 h without stirring. The product is collected by centrifugation (7000 rpm) and washed with ethanol for several times.

Synthesis of single-holed cobalt/N-doped carbon hollow particles. Typically, the above PS@ZIF-67 particles are re-dispersed into 1 ml of ethanol and placed in a ceramic boat, heated to 200 ^oC at ramp rate of 5 ^oC min^{-1} and nitrogen flow rate of 60 cm^3 min^{-1} for 2 h in a tube furnace (Lindberg/Blue M). The temperature in the furnace is further raised to 800 ^oC at a ramp rate of 5 ^oC min^{-1} and kept at that temperature for 2 h. After that, the furnace is cooled down to room temperature naturally.

Synthesis of MnO_x@ZIF-67 nanowires. The MnO_x nanowires are prepared by hydrothermal reaction (*Adv. Energy Mater.* **2016**, *6*, 1601177). Then the as-prepared MnO_x nanowires are dispersed in 10 ml of ethanol solution of PVP (0.5 g, M_w = 40000, Sigma-Aldrich), and the mixture is further stirred at room temperature for 12 h. The PVP-functionalized MnO_x nanowires are collected by centrifugation (8000 rpm), washed by ethanol several times, and dispersed in 15 ml of methanol for further use. To synthesize MnO_x@ZIF-67 nanowires, 0.3 ml of MnO_x nanowire suspension, 3 ml of 20 mM $Co(NO_3)_2·6H_2O$ (98%, Sigma-Aldrich) solution, and 5 ml of 160 mM 2-MIM (95%, Sigma-Aldrich) solution are mixed and then allowed to react at room temperature for 2 h without stirring. The product is then collected by centrifugation (7000 rpm) and washed with ethanol several times.

Synthesis of GO@ZIF-67 nanosheets. Graphene oxide (GO) nanosheets are prepared according to Hummers method (*J. Am. Chem. Soc.* **1958**, *80*, 1339). In a typical synthesis, 0.05 g of the GO nanosheet is dispersed in 10 ml of ethanol solution of PVP (0.5 g, M_w = 40000, Sigma-Aldrich), and the mixture is further stirred at room temperature for 12 h. The PVP-functionalized GO nanosheets are collected by centrifugation (10000 rpm), washed by ethanol several times, and dispersed in 10 ml of methanol for further use. To synthesize GO@ZIF-67 nanosheets, 0.1 ml of GO suspension, 3 ml of 20 mM $Co(NO_3)_2 \cdot 6H_2O$ (98%, Sigma-Aldrich) solution, and 5 ml of 160 mM 2-MIM (95%, Sigma-Aldrich) solution are mixed and then allowed to react at room temperature for 2 h without stirring. The product is then collected by centrifugation (7000 rpm) and washed with ethanol several times.

Materials characterization. Field-emission scanning electron microscope (FESEM; JEOL-6700F) and transmission electron microscope (TEM; JEOL, JEM-2010) are used to examine the morphology and structure of the samples. The composition of the sample is analyzed by energy-dispersive X-ray spectroscopy (EDX) attached to the FESEM instrument. The crystal phase of the products is examined by X-ray diffraction (XRD) on a Bruker D2 Phaser X-Ray Diffractometer.

Electrochemical measurements. The electrochemical measurements are carried out on glassy carbon disk with 5 mm in diameter that is polished with 0.05 micron alumina paste and then sonicated in distilled water and ethanol. Catalyst inks are prepared by dispersing 1 mg of catalyst materials in the mixture of isopropanol (0.2 ml) and Nafion (5 wt.%, 10 μl). The ink solution is then sonicated for 30 min to get a uniform suspension. Then, 10 μl of the catalyst ink is deposited on the glassy carbon substrate to obtain a loading of 0.25 mg cm^{-2}. Platinum plate (1 cm^2) and saturated calomel electrode (SCE) are selected as the counter electrode and reference electrode, respectively. All potentials in this study are measured against the SCE reference electrode and converted to the

reversible hydrogen electrode (RHE) reference scale by $E_{(RHE)} = E_{(SCE)} + 0.059pH + 0.242$. The electrochemical measurements are carried out at room temperature in a conventional three-electrode cell with 0.1 M KOH solution using a rotating disk electrode (RDE) setup from Pine Instrument Company connected to an Autolab potentiostat/galvanostat (Model PGSTAT-72637) workstation. The working electrode is scanned in 0.1 M KOH solution by CV at 20 mV s^{-1} and linear sweep voltammetry (LSV) at 10 mV s^{-1}. Before CV and RDE tests, the 0.1 M KOH solution is bubbled with oxygen for 30 min. The current densities in both CV and RDE data are normalized to the geometric area (0.196 cm^2) of the glassy carbon disk.

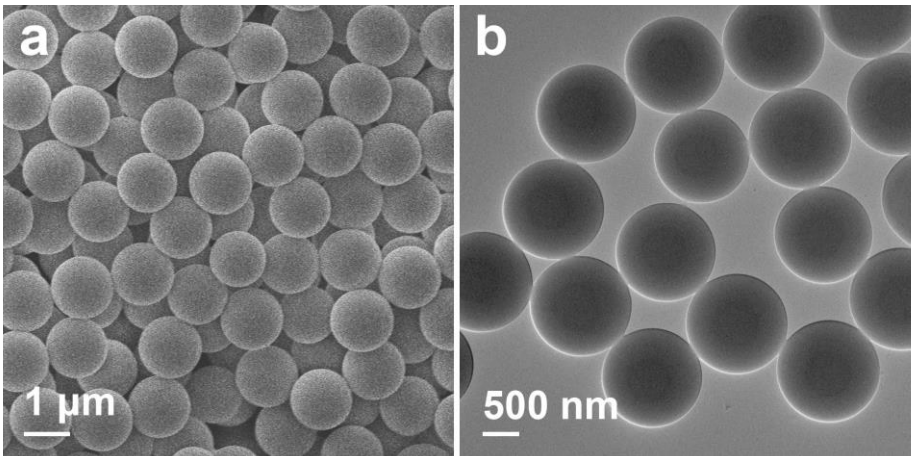

Figure S1. FESEM (a) and TEM (b) images of PVP functionalized PS spheres.

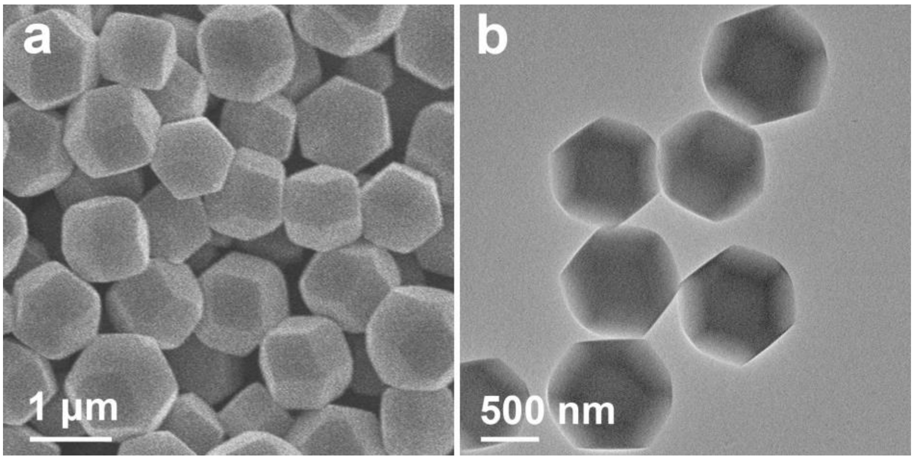

Figure S2. FESEM (a) and TEM (b) images of ZIF-67 particles.

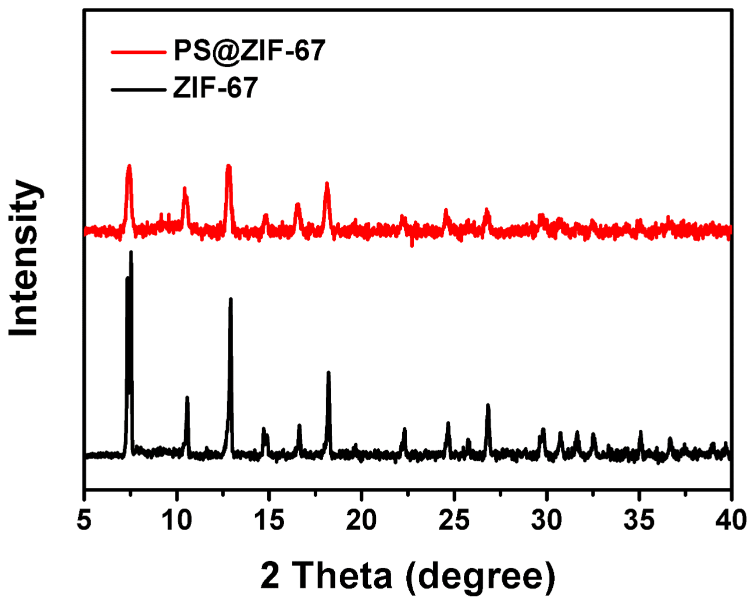

Figure S3. XRD patterns of PS@ZIF-67 and ZIF-67 particles.

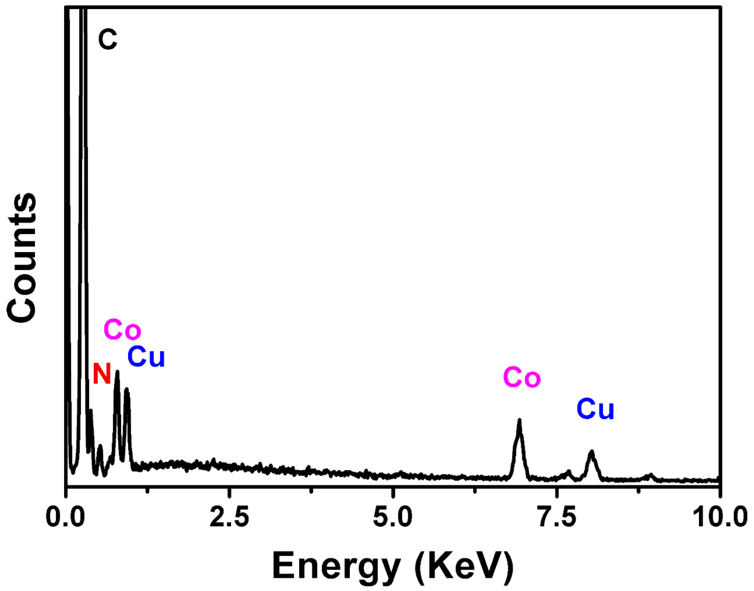

Figure S4. EDX spectrum of PS@ZIF-67 particles.

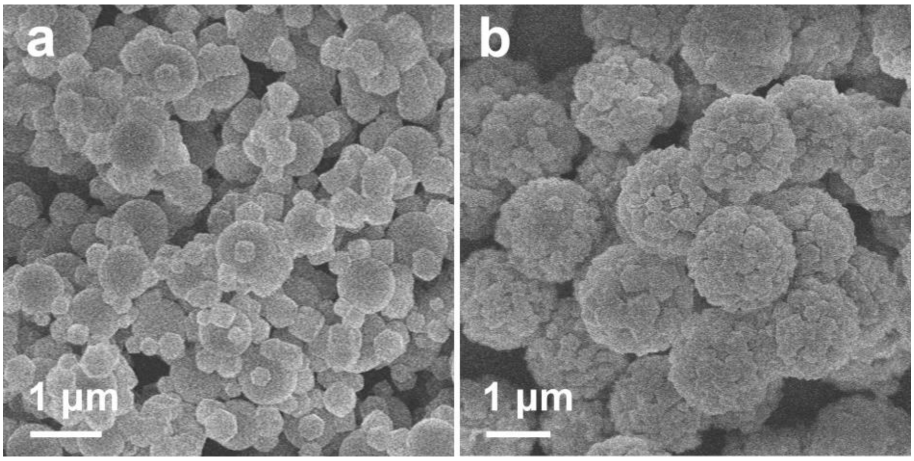

Figure S5. FESEM images of the PS@ZIF-67 composite particles prepared with different concentration of 2-MIM: 80 mM (a) and 120 mM (b).

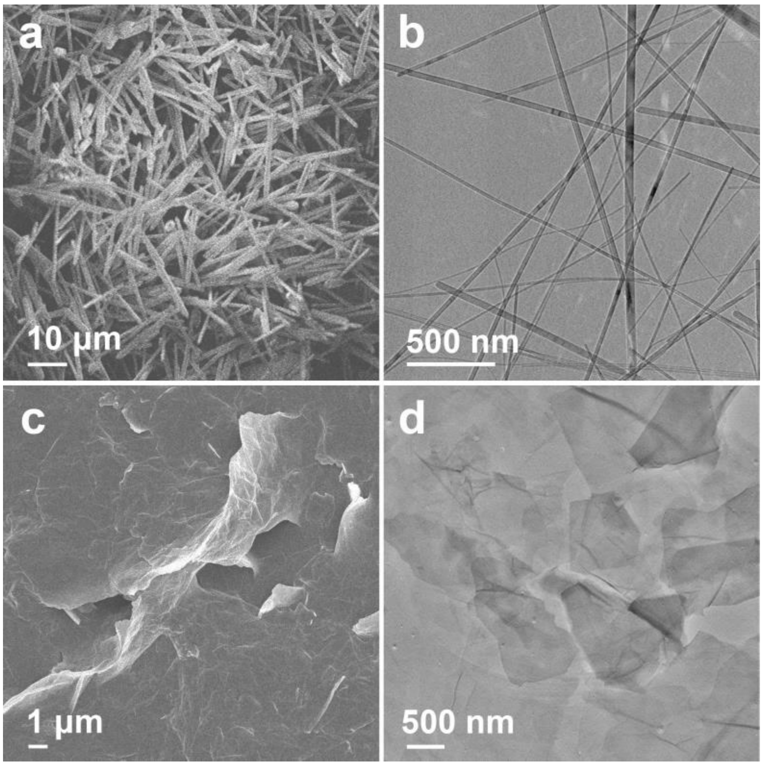

Figure S6. FESEM (a,c) and TEM (b,d) images of MnO_x nanowires (a,b) and GO nanosheets (c,d).

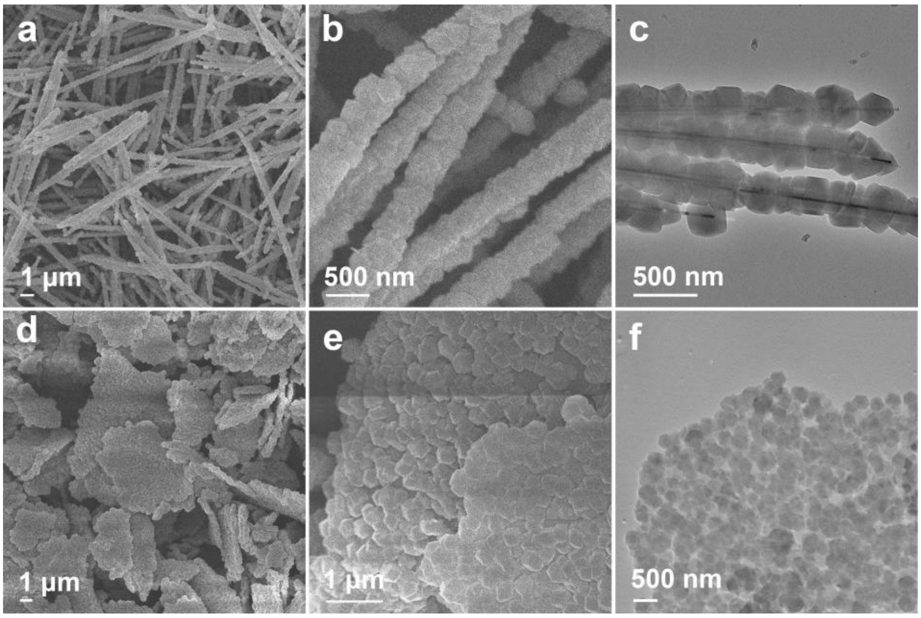

Figure S7. FESEM (a,b,d,e) and TEM (c,f) images of MnO_x@ZIF-67 nanowires (a-c) and GO@ZIF-67 nanosheets (d-f).

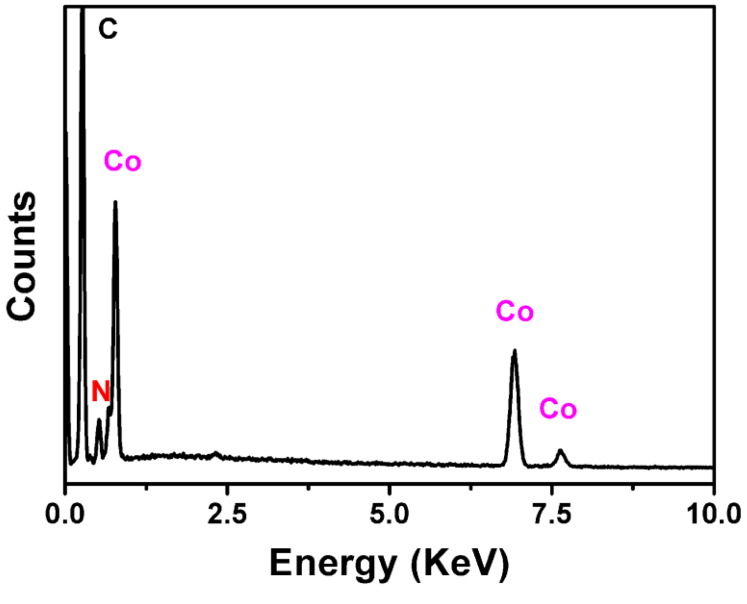

Figure S8. EDX spectrum of single-holed Co/NC hollow particles.

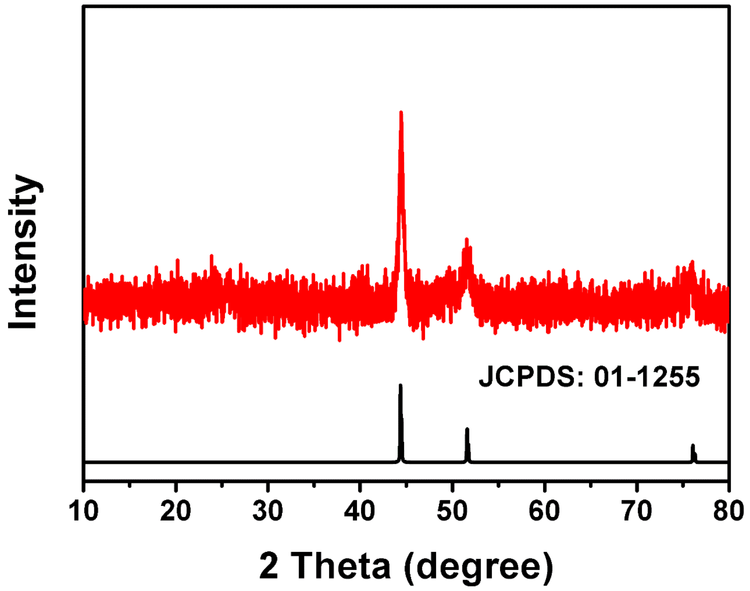

Figure S9. XRD pattern of single-holed Co/NC hollow particles.

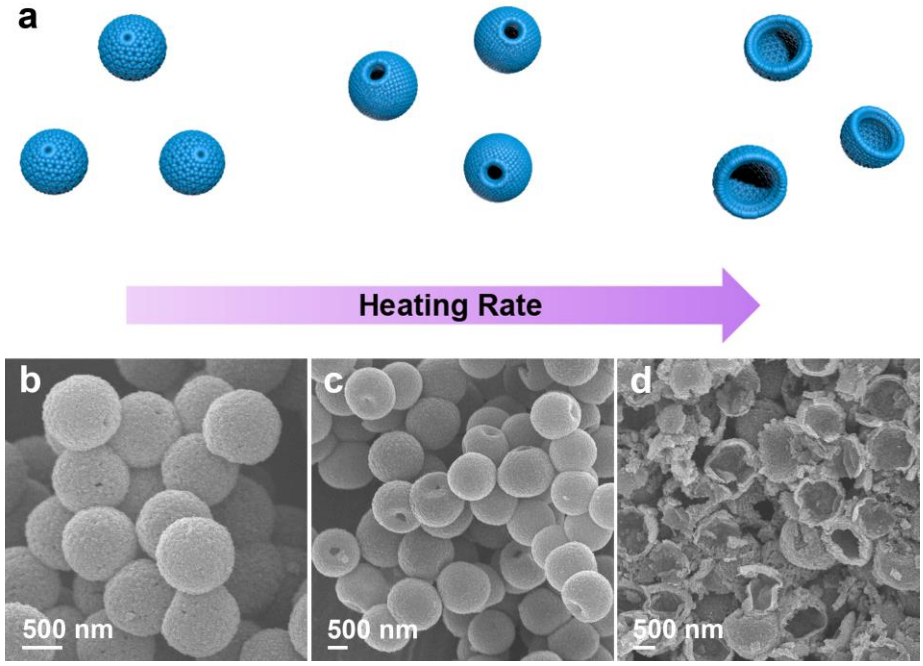

Figure S10. Schematic representation of the formation of Co/NC particles with different architectures (a). FESEM images of the PS@ZIF-67 particles-derived Co/NC particles prepared with different heating rates: 1 $^{o}C\ min^{-1}$ (b), 5 $^{o}C\ min^{-1}$ (c), and 10 $^{o}C\ min^{-1}$ (d).

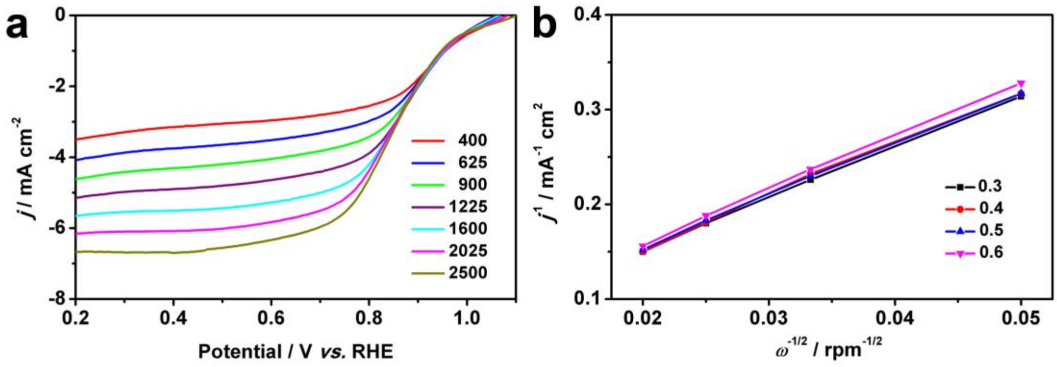

Figure S11. (a) LSV curves of single-holed Co/NC particles in O_2-saturated 0.1 M KOH solution with a sweep rate of 10 mV s^{-1} at different rotating speeds ranging from 400 to 2500 rpm. (b) Corresponding Koutecky-Levich plots (j^{-1} *vs*. $\omega^{-1/2}$) at different potentials from 0.3 to 0.6 V from the LSV curves shown in (a).

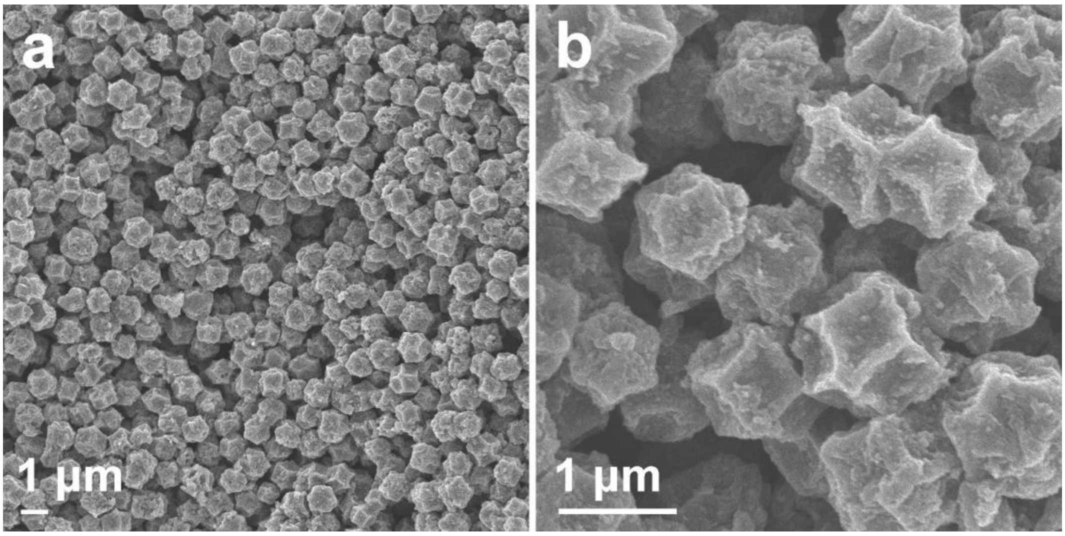

Figure S12. FESEM images of simple Co/NC particles derived from ZIF-67 particles.

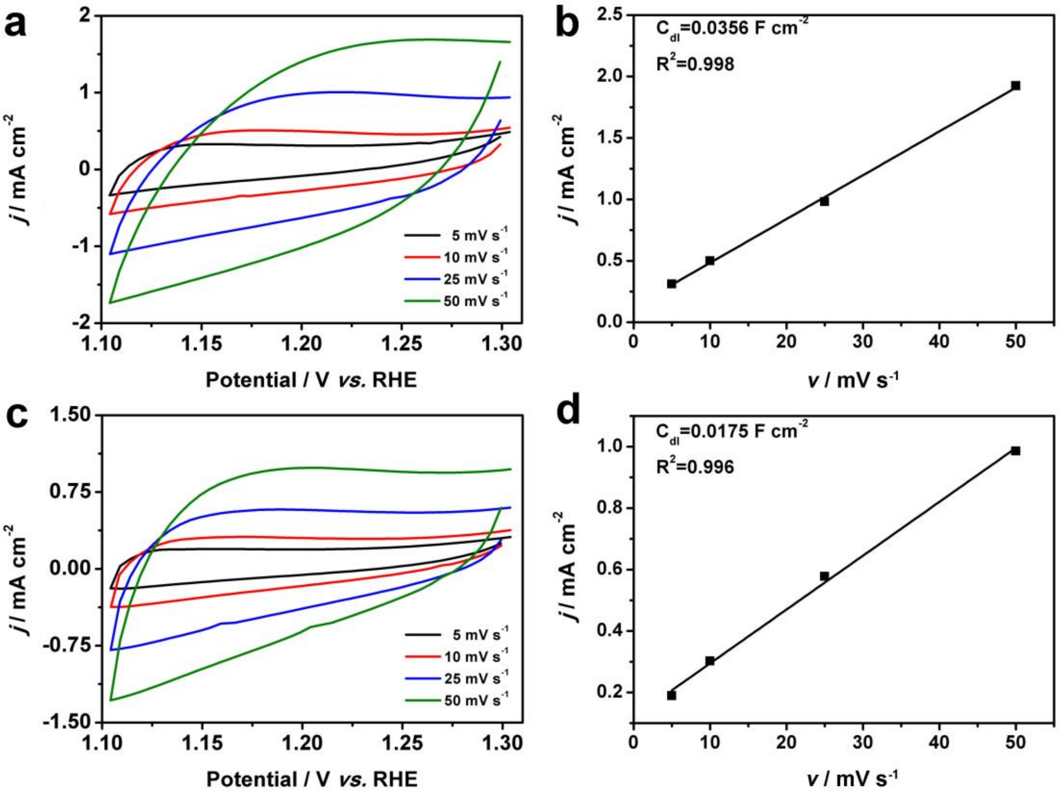

Figure S13. CV curves of (a) single-holed Co/NC hollow particles and (c) simple Co/NC particles in the double layer region at scan rates of 5, 10, 25, and 50 mV s^{-1} in 0.1 M KOH aqueous electrolyte; (b) and (d) current density (taken at the potential of 1.20 V) as a function of scan rate derived from (a) and (c), respectively.

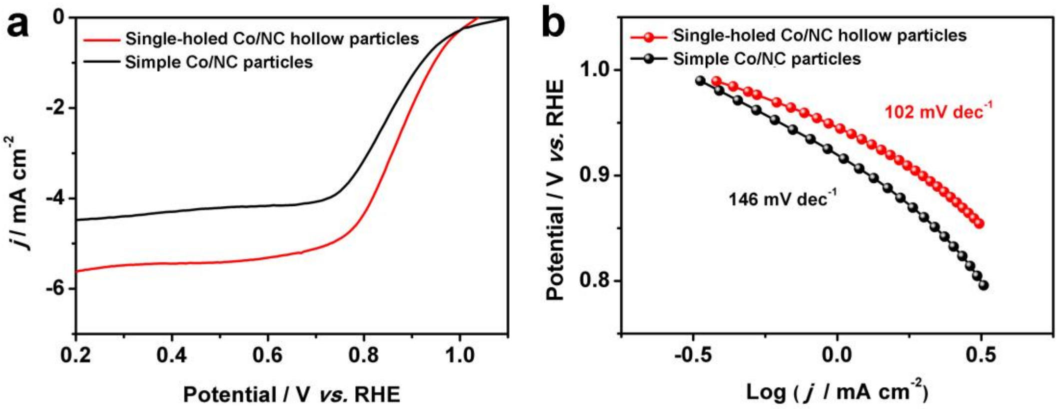

Figure S14. (a) LSV curves and (b) Tafel plots of single-holed Co/NC hollow particles and simple Co/NC particles.

**Table S1.** Summary of various MOF-derived electrocatalysts for ORR.					
**Catalyst**	**Onset potential (V)**	**Half-wave potential (V)**	**CV peak potential (V)**	**n**	**Reference**
N-CNT frameworks	0.97	0.87	0.87	3.97	*Nat. Energy* **2016**, *1*, 15006
Fe_3C@N-CNT assemblies	0.97	0.85	0.83	3.96	*Energy Environ. Sci.*, **2016**, *9*, 3092
N-doped nanoporous carbon	1.02	0.90	0.83	4.00	*Adv. Funct. Mater.* **2017**, *27*, 1606190
N-doped micro/mesoporous carbon	0.88	0.82	N/A	3.73	*Adv. Funct. Mater.* **2016**, *26*, 8334
$Ni_xCo_yO_4$/Co-NG	N/A	0.80	N/A	3.98	*J. Mater. Chem. A*, **2017**, *5*, 5594
Co-N-doped CFs	N/A	0.83	0.81	3.97	*J. Mater. Chem. A*, **2017**, *5*, 1211
NC@GC	1.00	0.93	N/A	3.98	*Nano Energy* **2016**, *30*, 368
C_3N_4@NH_2-MIL-101-700	0.99	0.84	N/A	3.70	*ACS Appl. Mater. Interfaces* **2016**, *8*, 35281
DCI-Fe-700	N/A	0.81	0.78	3.67	*ACS Appl. Mater. Interfaces* **2017**, *9*, 5272
Co/CoN_x/N-CNT/C	0.90	0.80	0.77	3.77	*ACS Appl. Mater. Interfaces* **2017**, *9*, 2541
Single-holed Co/NC hollow particles	0.98	0.87	0.83	3.99	This work
